# Natural borneol sensitizes human glioma cells to cisplatin-induced apoptosis by triggering ROS-mediated oxidative damage and regulation of MAPKs and PI3K/AKT pathway

**DOI:** 10.1080/13880209.2019.1703756

**Published:** 2019-12-26

**Authors:** Wen-qiang Cao, Xiao-qian Zhai, Ji-wei Ma, Xue-qi Fu, Bai-song Zhao, Pu Zhang, Xiao-yan Fu

**Affiliations:** a School of Life Sciences, Jilin University, Changchun, China; b Department of Biotechnology, Zhuhai Hopegenes Medical & Phamaceutical Institute, Zhuhai, China; c Department of Pathology, The Second Affiliated Hospital of Shandong First Medical University, Taian, China; d Department of Pathology, Shandong Provincial Hospital Affiliated to Shandong University, Jinan, China; e Department of Cardiology, The Central Hospital of Taian, Taian, China; f School of Basic Medicine, Shandong First Medical University & Shandong Academy of Medical Sciences, Taian, China

**Keywords:** Chemo-sensitization, DNA damage, reactive oxygen species, p53, ERK, Akt

## Abstract

**Context:**

Cisplatin-based chemotherapy was widely used in treating human malignancies. However, side effects and chemoresistance remains the major obstacle.

**Objective:**

To verify whether natural borneol (NB) can enhance cisplatin-induced glioma cell apoptosis and explore the mechanism.

**Materials and methods:**

Cytotoxicity of cisplatin and/or NB towards U251 and U87 cells were determined with the MTT assay. Cells were treated with 0.25–80 μg/mL cisplatin and/or 5–80 μM NB for 48 h. The effects of NB and/or cisplatin on apoptosis and cell cycle distribution were quantified by flow cytometric analysis. Protein expression was detected by western blotting. ROS generation was conducted by measuring and visualising an oxidation-sensitive fluorescein DCFH-DA.

**Results:**

NB synergistically enhanced the anticancer efficacy of cisplatin in human glioma cells. Co-treatment of 40 μg/mL NB and 40 μg/mL cisplatin significantly inhibited U251 cell viability from 100% to 28.2% and increased the sub-G1 population from 1.4% to 59.3%. Further detection revealed that NB enhanced cisplatin-induced apoptosis by activating caspases and triggering reactive oxygen species (ROS) overproduction as evidenced by the enhancement of green fluorescence intensity from 265% to 645%. ROS-mediated DNA damage was observed as reflected by the activation of ATM/ATR, p53 and histone. Moreover, MAPKs and PI3K/AKT pathways also contributed to co-treatment-induced U251 cell growth inhibition. ROS inhibition by antioxidants effectively improved MAPKs and PI3K/AKT functions and cell viability, indicating that NB enhanced cisplatin-induced cell growth in a ROS-dependent manner.

**Discussion and conclusions:**

Natural borneol had the potential to sensitise human glioma cells to cisplatin-induced apoptosis with potential application in the clinic.

## Introduction

Cisplatin, a traditional chemotherapeutic drug, has been widely applied in treating human solid malignancies, including nasopharyngeal, oesophageal, and lung carcinoma, ovarian cancer, and glioma (Rocha et al. 2015; Gong et al. 2018; Okamoto et al. 2018). Cisplatin can interact with DNA and activate apoptotic signalling transduction, which is also negatively regulated by prosurvival factors, such as Bcl-2 family members, PI3K/Akt, and various drug efflux pumps (e.g., ABCB1, also named P-gp or MDR1) (Wijdeven et al. 2016; Lipinska et al. 2017). Considering the development of drug resistance, patients have to receive high doses of cisplatin, thereby leading to undesirable side effects (Sprauten et al. 2012). Chemo-sensitization is also an effective strategy to overcome drug resistance and reduce adverse effects (Cao et al. 2014; Zhang et al. 2014; Gong et al. 2018). Thus, the development of novel chemosensitizers to enhance the anticancer efficacy of cisplatin-based therapy is important in clinical cancer treatment.

Borneol (C_10_H_18_O) is a bicyclic monoterpene extracted from the resin and volatile oil of dipterocarp (Wu et al. 2016). Borneol has been broadly applied in food and drug industry and is available as synthetic (SB) and natural borneol (NB) (Qi et al. 2013; Wu et al. 2016). Considering the mucosa stimulus and hepatotoxicity of SB, NB has more potential for further commercial applications. NB effectively improves the permeability of drugs by regulating intestinal mucous or blood–brain barrier (Cai et al. 2008; Duan et al. 2016; Xing et al. 2016). NB can also enhance the efficacy of chemo-drugs against various cancer cell lines by increasing the intracellular drug accumulation and modulating ROS-mediated DNA damage with the involvement of MAPKs activation and Akt inactivation (Chen, Li Su, and Chen 2015; Chen, Li, Su, Li, et al. 2015; Liu et al. 2018; Meng et al. 2018). The action of NB in chemo-sensitization is constantly being confirmed. Nevertheless, data on the synergistic effects of NB and cisplatin in human glioma treatment are limited. Therefore, the present study aims to verify whether NB plays a role in cisplatin-induced glioma cell apoptosis. The underlying mechanisms were also illuminated by the detection of ROS generation and related protein expressions.

## Materials and methods

### Reagents

NB (purity, 99%) was obtained from Guangzhou New BenFu Technology (Guangzhou, China). Bicinchoninic acid (BCA) kit was purchased from Beyotime Biotechnology (Shanghai, China). Dulbecco’s modified Eagle’s medium (DMEM), foetal bovine serum (FBS) and penicillin-streptomycin were purchased from Gibco (Shanghai, China). Cisplatin, thiazolyl blue tetrazolium bromide (MTT), propidium iodide (PI), glutathione (GSH) and 2′,7′-dichlorofluorescein diacetate (DCFH-DA) were purchased from Sigma-Aldrich (St. Louis, MO, USA). The specific inhibitors of MAPKs (i.e., SP600125, SB203580, and U0126) and PI3K (i.e., LY294002) and all the antibodies were purchased from Cell Signalling Technology Inc (Beverly, MA, USA).

### Cell culture

Human U87 and U251 glioma cells and HUVECs human umbilical vein endothelial cells were obtained from American Type Culture Collection (Manassas, VA). U87 and U251 cells were cultured in DMEM medium supplemented with 10% of FBS, 100 units/mL of penicillin and 50 units/mL of streptomycin. HUVECs were maintained in endothelial cell growth medium supplemented with foetal bovine serum (10%), penicillin (100 units/mL), and streptomycin (50 units/mL). All these cells were stored in a humidified incubator with 5% CO_2_ atmosphere at 37 °C.

### Drug treatment and cell viability detection

The U87 and U251 cells (6 × 10^3^ cells/well) cultured in 96-well plate were exposed to 2.5–80 μg/mL cisplatin and/or 5–80 μg/mL NB for 48 h. After treatment, cell viability was determined by MTT assay. Briefly, cells were incubated on the basis of the prescribed NB and cisplatin concentrations in 96-well plates. Then, MTT (20 μL, 5 mg/mL) was added and incubated for 5 h. The medium in wells was removed, and then 150 μL of DMSO was added into per well to dissolve the formazan salt formed. The colour intensity of the formazan solution was detected at 570 nm with a microplate reader (VERSA max).

### Cell cycle distribution and apoptosis detection

The effects of NB and/or cisplatin on apoptosis and cell cycle distribution were quantified by flow cytometric analysis. Briefly, cells after treatment were harvested and fixed with 70% ethanol overnight. After the incubation with PI for 3 h in dark, the stained cells were determined by flow cytometery and analysed using MultiCycle software. Hypodiploid DNA content (Sub-G1) was employed to quantify the apoptotic cells. 10000 cells per sample were examined.

### Intracellular ROS generation

The detection of ROS generation was conducted by measuring and visualising an oxidation-sensitive fluorescein DCFH-DA. The untreated cells were harvested, washed with PBS two times, and treated with DCFH-DA (10 μM, 37 °C) for 30 min. Then cells were treated with NB and/or cisplatin for 6 h. After the treatment, ROS generation was examined by a microplate reader and a fluorescence microscope (488 nm, 525 nm). Data were expressed as % of control.

### Western blotting

The treated U251 cells were washed with PBS and then incubated with cell lysis buffer overnight at −20 °C. Protein electrophoresis and blotting were performed as described in our previous methods (Cao et al. 2014). Briefly, SDS-PAGE was done in 12% Tricine gels loading 30 μg of cell lysates per lane. After electrophoresis, the separated proteins were transferred to nitrocellulose membrane for 75 min at 110 V and blocked with 5% non-fat milk in TBS buffer for 1 h. Then, the membrane was washed with TBST buffer and incubated with primary antibodies (1:1000) overnight at 4 °C and secondary antibodies (1:2000) for 2 h at room temperature. The targeted proteins were displayed on X-ray film by using an enhanced chemiluminescence reagent. β-actin was used to confirm the equal loading and transfer of proteins. The protein expression was quantified by Quantity-One Software. Changes in the levels of protein expression were expressed as the percentage (%) of control group.

### Statistical analysis

All data were derived from three independent experiments and displayed as mean ± standard deviation. Statistical analysis was conducted using SPSS 13.0 software. Difference between two groups was analysed by two-tailed Student’s *t*-test. The significance among three or more groups was analysed by multiple comparisons. The bars * and ** represent *p* < 0.05 and *p* < 0.01, respectively. Bar with different letters indicate the statistical significance at *p* < 0.05 level.

## Results

### NB synergistically enhanced cisplatin-induced human glioma cell death

**Figure 1.**
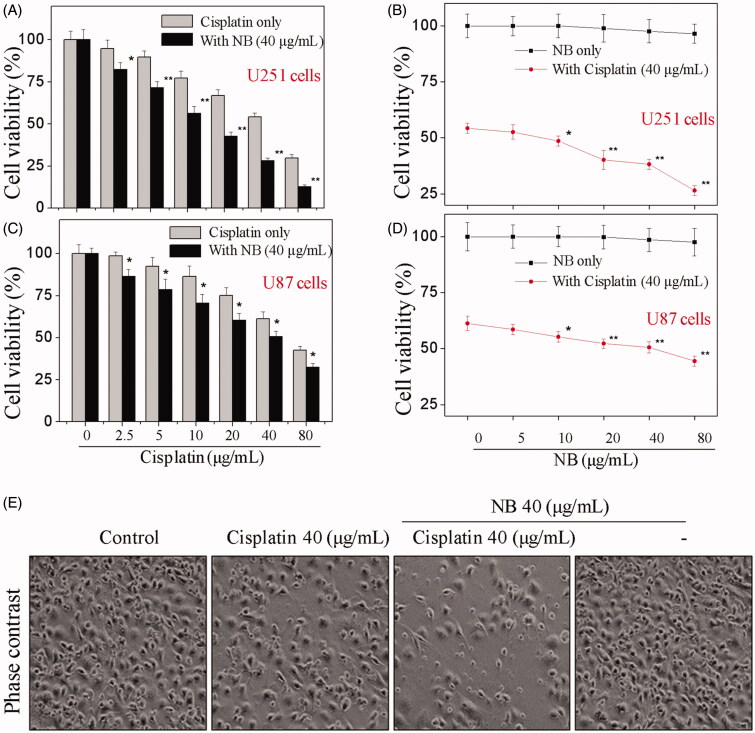
NB enhances cisplatin-induced cytotoxicity in human glioma cells. (A-D) Cytotoxicity of cisplatin and NB towards U251 and U87 cells. Cells were treated with 0.25–80 μg/mL cisplatin and/or 5–80 μg/mL NB and cell viability was determined by MTT assay after 48 h treatment. Each value represents the mean ± SD of three independent experiments. Bars with “*” or “**” indicate the *p* < 0.05 or *p* < 0.01, respectively. (E) Changes in the morphology of U251 cells after indicated treatments as visualised by phase contrast microscope (magnification, 200×).

In the present work, human glioma U87 and U251 cells were selected to evaluate the growth inhibitory effects of cisplatin and/or NB. As shown in Figure 1(A,C), the treatment of U87 and U251 cells with 2.5–80 μg/mL cisplatin for 48 h significantly suppressed cell proliferation. Inconsistent with cisplatin treatment, results in Figure 1(B,D) showed that the changes in cell viability were minimal after the exposure of 5–80 μg/mL NB for 48 h. For the combined treatment, cisplatin-mediated cell growth inhibition was significantly enhanced by the addition of 40 μg/mL NB (Figure 1(A,C)). The combination of 40 μg/mL cisplatin with 5–80 μg/mL NB also achieved synergistic effect against U87 and U251 cells. The decrement of cell populations and appearance of cell rounding and shrinkage were also visualised by a phase-contrast microscope after cisplatin and/or NB treatment (Figure 1(E)). Despite the remarkable antiproliferative effects, the co-treatment also showed lower cytotoxicity towards HUVECs human umbilical vein endothelial cells (Figure S1). The above evidence showed that NB synergistically enhanced the anticancer efficacy of cisplatin in human glioma cells. Due to the more sensitive of U251 cells to NB and/or DOX, U251 cell line was selected to further evaluate the underlying mechanisms of the co-treatment.

### NB potentiated cisplatin-induced U251 cell apoptosis with the involvement of caspases activation

**Figure 2.**
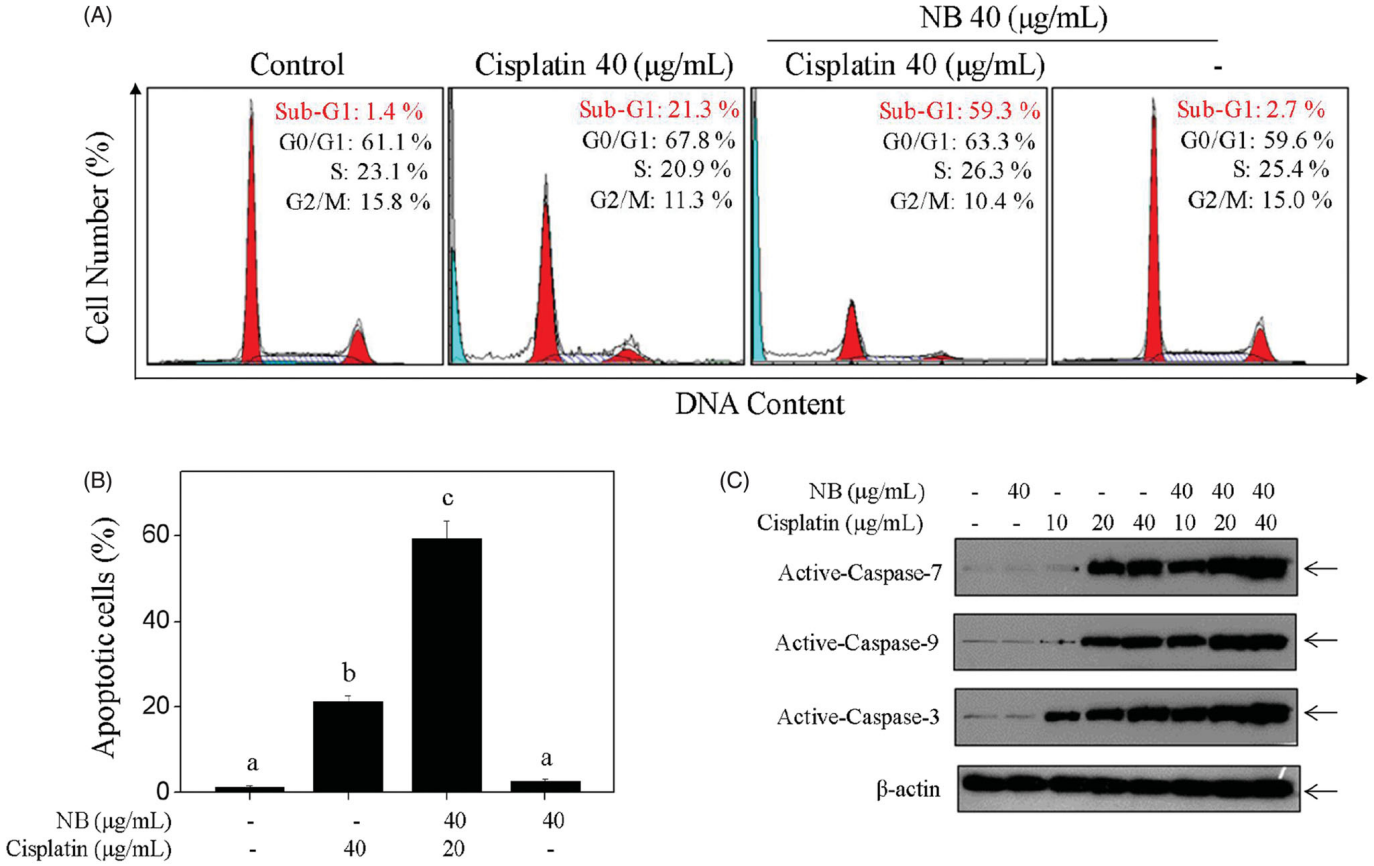
NB potentiates cisplatin-induced U251 cells apoptosis with involvement of caspases activation. (A, B) Quantitative analysis of cell cycle distribution and apoptotic cell death were measured by flow cytometric analysis. Cells were treated with 40 μg/mL NB and/or 40 μg/mL cisplatin for 48 h. (C) Western blot analysis of active-caspase −3, −7, −9 in U251 cells after the treatment of 40 μg/mL NB and/or 40 μg/mL cisplatin for 48 h. Each value represents the mean ± SD of three independent experiments. Bars with different characters (a, b, c and d) are statistically different at *p* < 0.05 level.

To validate the mechanisms of co-treatment-mediated synergistic anticancer effects, we conducted flow cytometric analysis to analyse the alternation of cell cycle progression and apoptosis. As shown in Figure 2(A,B), the combined treatment of cisplatin and NB resulted in a notable increase in the number of apoptotic cells in comparison with the single treatment, as evidenced by the sub-diploid peaks. For example, cisplatin-mediated the accumulation of sub-G1 population was further increased from 21.3% to 59.3% by the addition of 40 µg/mL of NB for 48 h. Moreover, little alternation in cell cycle progression was observed in single or combined treatment. Hoechst33342/PI co-staining assay further confirmed co-treatment-induced U251 cells apoptosis, not necrosis (Figure S2). Therefore, these results indicated that the co-treatment-mediated U251 cell growth inhibition was mainly caused by induction of apoptosis.

Generally, apoptosis is initiated by two major mechanisms, namely, caspase-8-mediated extrinsic and caspase-9-mediated intrinsic pathways. To prove the findings mentioned above, we determined the activation of caspase family members by western blotting. As shown in Figure 2(C), S3, the activated caspase −3, −7, and −9 were moderately increased with the treatment of 10, 20, and 40 µg/mL of cisplatin, respectively. In contrast to single treatment, the activation of caspase −3, −7, and −9 was further enhanced after the combination of 40 µg/mL of NB, which indicated the involvement of intrinsic apoptosis in co-treatment-induced U251 cell death.

### NB amplified cisplatin-induced ROS generation and DNA damage signalling transduction

**Figure 3.**
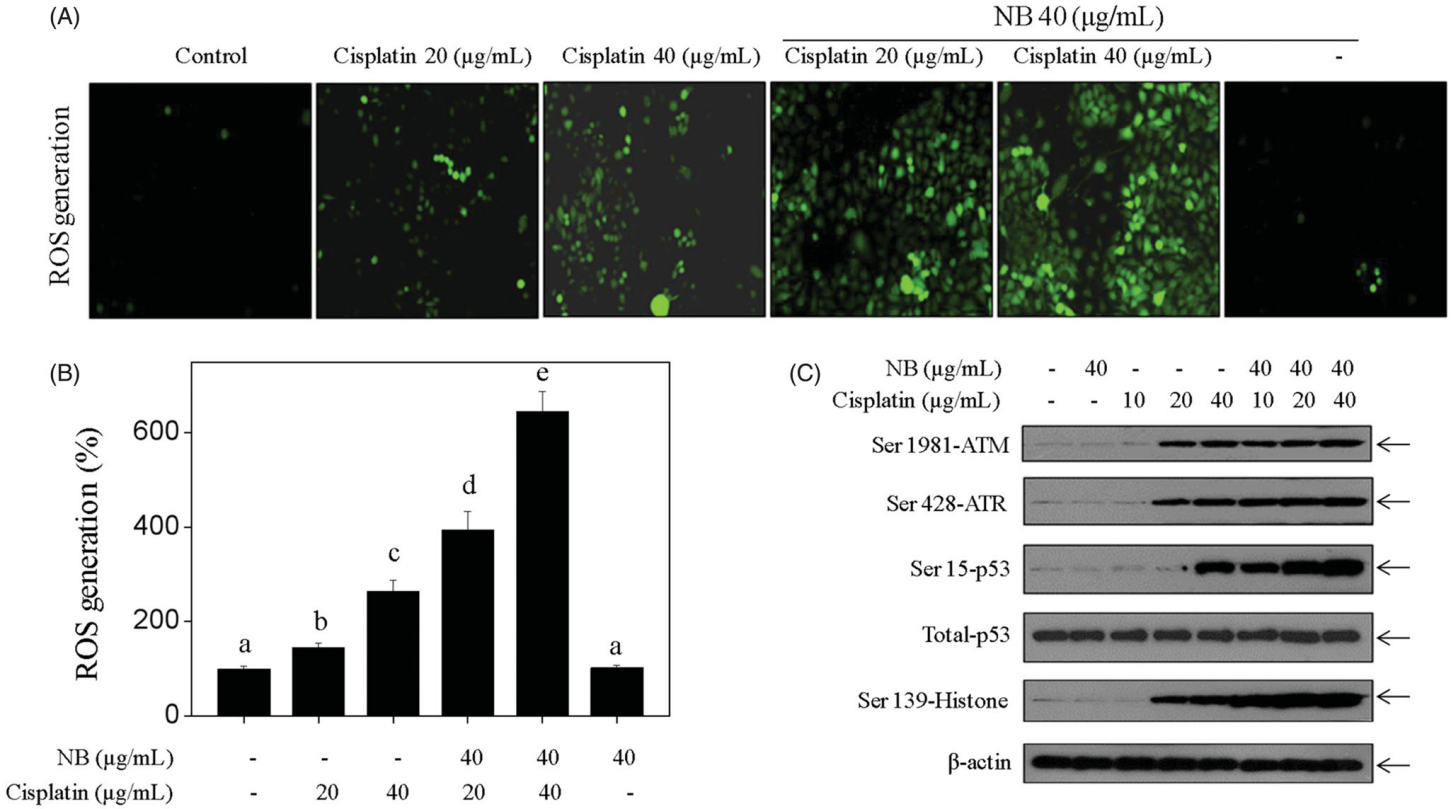
NB amplifies cisplatin-induced ROS generation and DNA damage-mediated signalling transduction in U251 cells. (A, B) NB and/or cisplatin-induced ROS accumulation in U251 cells. Cells were treated with NB (40 μg/mL) and/or indicated concentrations of cisplatin for 6 h. The levels of intracellular ROS were determined by DCF assay and was detected by visualising and measuring DCFH-DA. (C) Western blot analysis of expression levels of phosphorylated ATM, ATR, p53 and Histone H2A.X in U251 cells exposed to 40 μg/mL NB and/or cisplatin (10, 20, 40 μg/mL) for 48 h.

ROS production was detected by measuring and visualising an oxidation-sensitive fluorescein DCFH-DA. Figure 3(A,B) showed that the intracellular ROS level was moderately enhanced by cisplatin treatment alone and further improved by co-treatment, thereby indicating the important role of NB in co-treatment-mediated ROS accumulation. Moreover, the expression levels of DNA damage-related mediators were detected by western blotting. As shown in Figure 3(C), S4, NB treatment significantly enhanced cisplatin-induced activation of ATM, ATR, p53, and histone H2A.X. These results suggested that NB had the potential to enhance cisplatin-induced DNA damage by ROS overproduction.

### Contributions of MAPKs and PI3K/Akt signalling pathways

**Figure 4.**
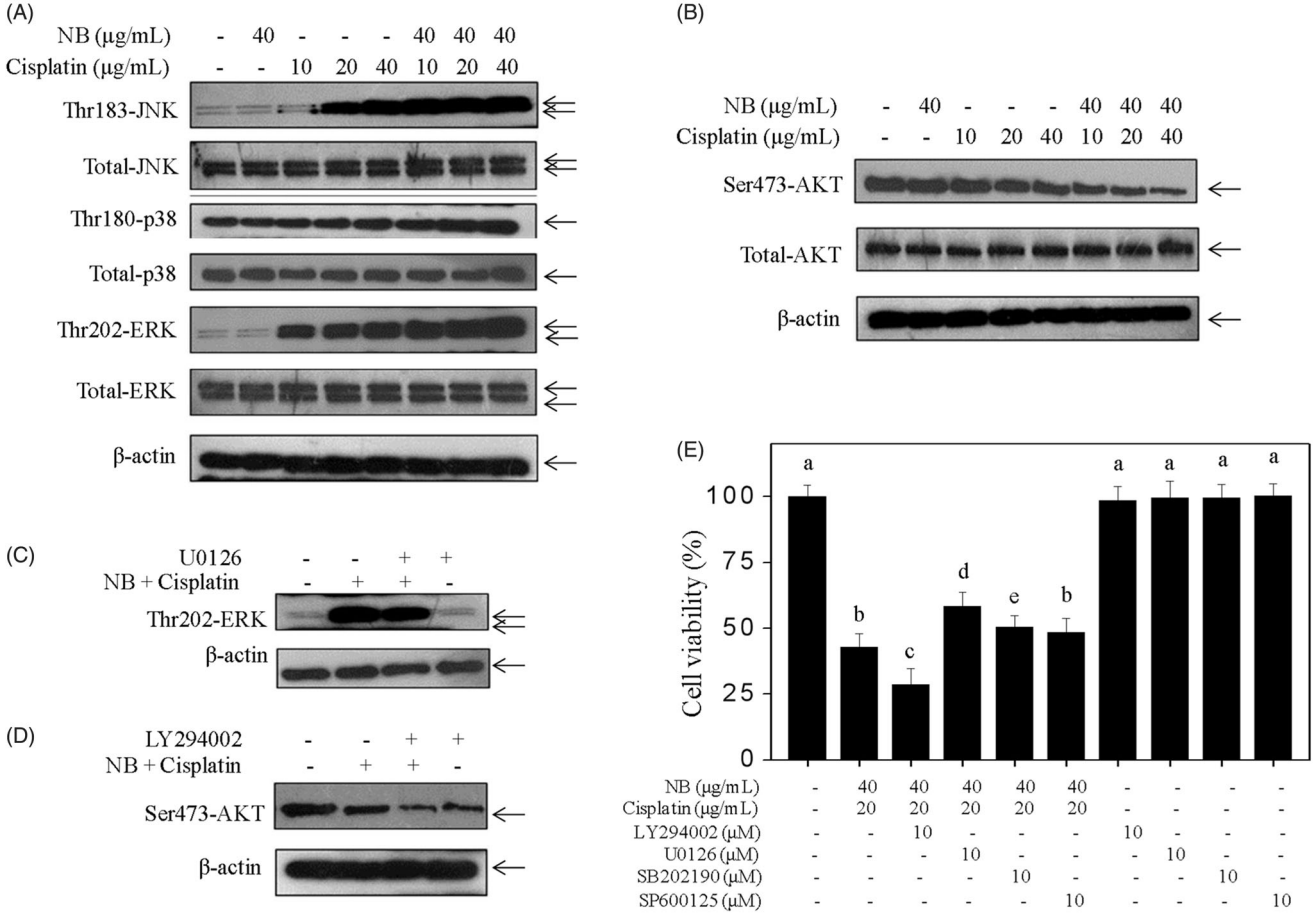
Effects of co-treatment on MAPKs and Akt signalling pathways. (A, B) Western bolt analysis of the phosphorylation status and the expression level of MAPKs and Akt. Cells were treated with indicated concentrations of NB and/or cisplatin for 48 h. (C, D) Effects of U0126 (ERK inhibitor) and LY294002 (PI3K inhibitor) on co-treatment-induced alternation of phosphorylation status of ERK and Akt in U251 cells. Cells were pre-treated with 10 µM LY294002 or U0126 for 1 h, and co-treated with 40 μg/mL NB and 40 μg/mL cisplatin for 48 h. (E) Effects of SP600125 (JNK inhibitor), SB203580 (p38 MAPK inhibitor), U0126 (ERK inhibitor) and LY294002 (PI3K inhibitor) on co-treatment-induced cell growth inhibition. Each value represents the mean ± SD of three independent experiments. Bars with different characters (a, b, c, d and e) are statistically different at *p* < 0.05 level.

MAPKs and PI3K/Akt both regulate cell growth by balancing the phosphorylated JNK, ERK, P38, and Akt levels. Results in the present study showed that the combined treatment of cisplatin and NB significantly activated the phosphorylation of JNK, P38, and ERK (Figure 4(A), S5) and suppressed Akt phosphorylation (Figure 4(B), S5) in contrast with the single treatment. The alternation in the total proteins of MAPKs and Akt was insignificant after treatment with cisplatin and/or NB. To confirm the role of MAPK and Akt phosphorylation, we detected the effects of specific inhibitors (i.e., SP600125, SB203580, U0126, and LY294002) on the co-treatment. As shown in Figure 4(C–E), pre-treatment of cells with 10 µM U0126 (ERK inhibitor) or 10 µM LY294002 (PI3K inhibitor) moderately changed the co-treatment-induced ERK and Akt activation and cell growth inhibition. Nevertheless, SP600125 (JNK inhibitor) or SB203580 (p38 inhibitor) exhibited minimal inhibitory effect on co-treatment-induced cell death (Figure 4(E)). These results indicated that ERK and Akt both contributed to co-treatment-induced apoptosis.

### ROS inhibition attenuated co-treatment-induced apoptosis by modulation of MAPKs and Akt phosphorylation

**Figure 5.**
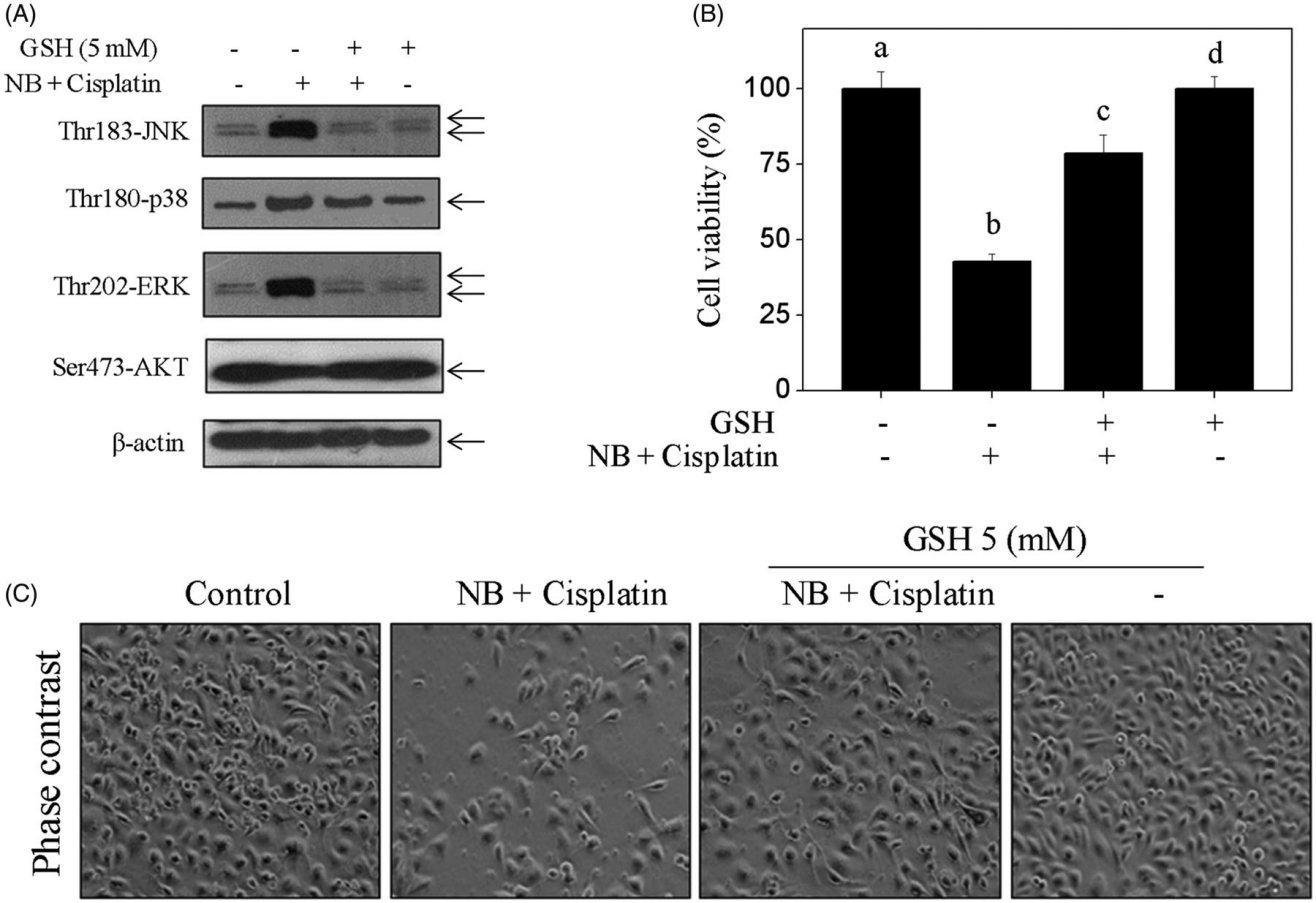
Antioxidants inhibits co-treatment-induced U251 cells apoptosis by changing MAPKs and Akt activation. (A) Protective effects of GSH on co-treatment-mediated JNK, p38, ERK and Akt phosphorylation. (B) Suppression of ROS by GSH reversed co-treatment-induced U251 cell death. Cells were pre-treated with 5 mM GSH for 1 h, and then co-treated with 40 μg/mL NB and 40 μg/mL cisplatin for 48 h. Cell viability and protein expression levels were measured as described in method section. Bars with different characters (a, b, c and d) are statistically different at *p* < 0.05 level. (C) Changes in the morphology of U251 cells after indicated treatments as visualised by phase contrast microscope (magnification, 200×).

In the present study, antioxidant (GSH) was introduced to detect whether ROS can regulate co-treatment-induced MAPK activation and Akt dephosphorylation in U251 cells. As shown in Figure 5(A), S7, pre-treatment of cells with 5 mM GSH significantly reduced co-treatment-induced MAPK activation and increased the phosphorylated status of Akt. The results in Figure 5(B,C) showed that co-treatment-induced U251 cell death was remarkably reversed by the addition of GSH. These results indicated that ROS, as an upstream initiator, regulates co-treatment-induced MAPKs activation and Akt dephosphorylation in U251 cells.

## Discussion

Human glioma is one of the most malignant and aggressive primary brain cancers with poor prognosis due to the development of drug resistance and undesirable BBB (Huse and Holland 2010; Schaff and Lassman 2015). As a broad-spectrum chemotherapeutic drug, the application of cisplatin in glioma treatment is still unsatisfactory. Considerable evidence showed that novel chemosensitizers could effectively improve the anticancer effects of cisplatin and reduce the undesirable adverse effects (Zhang et al. 2014; Gong et al. 2018; Wandee et al. 2019; Zhou et al. 2019). Recently, researchers have focussed on the synergistic properties of NB that can effectively improve cell permeability, open BBB, and accelerate drug distribution in tumour tissue (Cai et al. 2008; Duan et al. 2016; Xing et al. 2016). The synergistic potential of NB in chemo-sensitization is being concerned. In the present study, NB has been identified as a promising chemosensitzer that could synergistically enhance the anticancer efficacy of cisplatin in the treatment of human glioma.

Induction of apoptosis and cell cycle arrest are two main modes in which chemotherapeutic drugs inhibit tumour cell growth. Cisplatin is one of the most common chemodrugs that can inhibit cancer cell proliferation by inducing caspase-mediated apoptosis (Zhang et al. 2014). Previous studies showed the potential of NB in enhancing chemodrug-induced apoptosis (Chen, Li Su, and Chen 2015; Chen, Li, Su, Li, et al. 2015; Liu et al. 2018; Meng et al. 2018). Here, we provide convincing evidence that NB potentiated the anticancer efficacy of cisplatin through the induction of caspase-9-mediated cell apoptosis.

ROS is a critical upstream initiator in cellular signalling cascades, which modulate various cellular events, including cell growth, metastasis, and apoptosis (Apel and Hirt 2004; Pelicano et al. 2004). Previous studies have shown that the accumulation of intracellular ROS is closely related to NB-mediated chemo-sensitization (Zhang et al. 2014; Chen, Li Su, and Chen 2015; Liu et al. 2018). Herein, our data demonstrated that the combined treatment of cisplatin with NB remarkably increased the intracellular accumulation of ROS in comparison with single treatment. Excessive ROS generation regulates DNA damage-mediated signalling transduction, including the activation of ATM/ATR and various downstream substrates (i.e., H2AX and p53) to induce apoptosis (Lloyd et al. 1997). Results in Figure 3(C) showed that cisplatin-induced DNA damage and activation of downstream substrates (ATM/ATR, p53) were significantly enhanced by the introduction of NB. Taken together, these data indicated that ROS overproduction may play an important role in co-treatment-mediated U251 cell death.

In addition to the induction of ROS-mediated DNA damage, cisplatin-mediated cell death was regulated by MAPKs and PI3K/Akt pathways, which are major oxidative stress-sensitive kinases in most cancer cell types (Zhang et al. 2014; Wandee et al. 2019; Zhou et al. 2019). The abnormal activation of MAPKs (e.g., ERK1/2, JNK, and p38) and Akt is associated with the development of drug resistance. Thus, targeting MAPKs and Akt by chemosensitizer may improve the anticancer efficacy of cisplatin. Studies have shown that NB could remarkably improve the anticancer activities of paclitaxel, selenocysteine, and curcumin by activating MAPKs and suppressing Akt (Su et al. 2013; Chen et al. 2014; Chen, Li Su, and Chen 2015; Chen, Li, Su, Li, et al. 2015; Meng et al. 2018). The obtained data in the present study revealed that the abnormal activation of ERK and Akt contributed to co-treatment-induced apoptosis which was further confirmed by addition of specific inhibitors (U0126, LY294002). Researchers demonstrated that pre-treatment of antioxidant inhibited cisplatin- or NB-induced ROS generation and reversed ROS-mediated signalling transduction and cell death (Zhang et al. 2014). In accord with these data, our work showed that co-treatment-mediated abnormal activation of MAPK and Akt were significantly impacted by the pre-treatment of GSH. More importantly, co-treatment-induced U251 cell killing was notably suppressed by antioxidant, indicating ROS as an upstream initiator that regulates co-treatment-induced U251 cell death and activation of MAPKs and Akt.

## Conclusions

**Figure 6.**
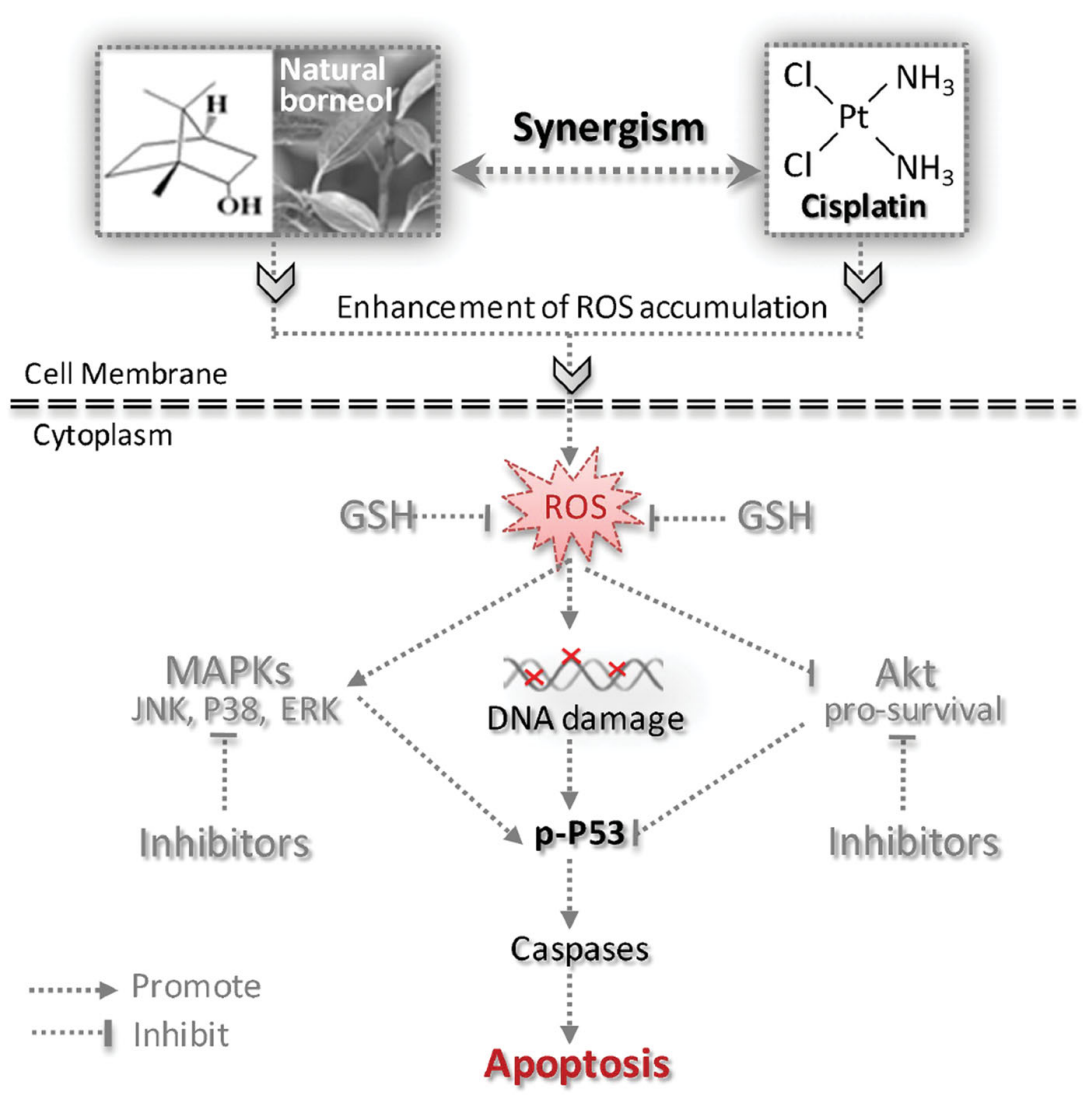
Proposed signalling pathway triggered by NB and cisplatin in U251 cells. NB synergistically enhances the anticancer efficacy of cisplatin through induction of ROS overproduction. The accumulation of intracellular ROS activates DNA damage-mediated downstream signalling transduction and regulates pro-apoptosis MAPK family members and pro-survival kinase Akt, leading to the obvious enhancement of apoptosis.

On the basis of the above data, we described a signalling pathway for the synergistic action of NB and cisplatin in Figure 6 **(**TOC Graphic). The combination of cisplatin and NB can be highly effective in achieving synergistic anticancer effect by triggering ROS-mediated oxidative damage and dysfunction of MAPKs and PI3K/Akt signalling pathways. Meanwhile, the combined strategy will be further confirmed by *in vivo* studies in the near future. NB may act as a promising apoptosis enhancer in cisplatin-based cancer treatment.

## Supplementary Material

Supplemental Material

## References

[CIT0001] Apel K, Hirt H. 2004. Reactive oxygen species: metabolism, oxidative stress, and signal transduction. Annu Rev Plant Biol. 55(1):373–399. 15377225 10.1146/annurev.arplant.55.031903.141701

[CIT0002] Cai Z, Hou S, Li Y, Zhao B, Yang Z, Xu S, Pu J. 2008. Effect of borneol on the distribution of gastrodin to the brain in mice via oral administration. J Drug Target. 16(2):178–184. 18274938 10.1080/10611860701794395

[CIT0003] Cao W, Li X, Zheng S, Zheng W, Wong YS, Chen T. 2014. Selenocysteine derivative overcomes TRAIL resistance in melanoma cells: evidence for ROS-dependent synergism and signaling crosstalk. Oncotarget. 5(17):7431–7445. 25277183 10.18632/oncotarget.2008 PMC4202134

[CIT0004] Chen J, Li L, Su J, Chen T. 2015. Natural borneol enhances bisdemethoxycurcumin-induced cell cycle arrest in the G2/M phase through up-regulation of intracellular ROS in HepG2 cells. Food Funct. 6(3):740–748. 25537301 10.1039/c4fo00807c

[CIT0005] Chen J, Li L, Su J, Li B, Chen T, Wong YS. 2014. Synergistic apoptosis-inducing effects on A375 human melanoma cells of natural borneol and curcumin. PLoS One. 9(6):e101277. 24971451 10.1371/journal.pone.0101277 PMC4074168

[CIT0006] Chen J, Li L, Su J, Li B, Zhang X, Chen T. 2015. Proteomic analysis of G2/M arrest triggered by natural borneol/curcumin in HepG2 cells, the importance of the reactive oxygen species-p53 pathway. J Agric Food Chem. 63(28):6440–6449. 26051007 10.1021/acs.jafc.5b01773

[CIT0007] Duan M, Xing Y, Guo J, Chen H, Zhang R. 2016. Borneol increases blood-tumour barrier permeability by regulating the expression levels of tight junction-associated proteins. Pharm Biol. 54(12):3009–3018. 27431008 10.1080/13880209.2016.1199044

[CIT0008] Gong S, Chen Y, Meng F, Zhang Y, Li C, Zhang G, Huan W, Wu F. 2018. Roflumilast enhances cisplatin-sensitivity and reverses cisplatin-resistance of ovarian cancer cells via cAMP/PKA/CREB-FtMt signalling axis. Cell Prolif. 51(5):e12474. 30069985 10.1111/cpr.12474 PMC6528923

[CIT0009] Huse JT, Holland EC. 2010. Targeting brain cancer: advances in the molecular pathology of malignant glioma and medulloblastoma. Nat Rev Cancer. 10(5):319–331. 20414201 10.1038/nrc2818

[CIT0010] Lipinska N, Romaniuk A, Paszel-Jaworska A, Toton E, Kopczynski P, Rubis B. 2017. Telomerase and drug resistance in cancer. Cell Mol Life Sci. 74(22):4121–4132. 28623509 10.1007/s00018-017-2573-2 PMC5641272

[CIT0011] Liu WJ, Yin YB, Sun JY, Feng S, Ma JK, Fu XY, Hou YJ, Yang MF, Sun BL, Fan CD. 2018. Natural borneol is a novel chemosensitizer that enhances temozolomide-induced anticancer efficiency against human glioma by triggering mitochondrial dysfunction and reactive oxide species-mediated oxidative damage. Onco Targets Ther. 11:5429–5439. 30233204 10.2147/OTT.S174498 PMC6129032

[CIT0012] Lloyd DR, Phillips DH, Carmichael PL. 1997. Generation of putative intrastrand cross-links and strand breaks in DNA by transition metal ion-mediated oxygen radical attack. Chem Res Toxicol. 10(4):393–400. 9114975 10.1021/tx960158q

[CIT0013] Meng X, Dong X, Wang W, Yang L, Zhang X, Li Y, Chen T, Ma H, Qi D, Su J. 2018. Natural borneol enhances paclitaxel-induced apoptosis of ESCC cells by inactivation of the PI3K/AKT. J Food Sci. 83(5):1436–1443. 29660811 10.1111/1750-3841.14143

[CIT0014] Okamoto T, Yano T, Shimokawa M, Takeo S, Yamazaki K, Sugio K, Takenoyama M, Nagashima A, Tsukamoto S, Hamatake M, et al. 2018. A phase II randomized trial of adjuvant chemotherapy with S-1 versus S-1 plus cisplatin for completely resected pathological stage II/IIIA non-small cell lung cancer. Lung Cancer. 124:255–259. 30268470 10.1016/j.lungcan.2018.08.015

[CIT0015] Pelicano H, Carney D, Huang P. 2004. ROS stress in cancer cells and therapeutic implications. Drug Resist Updat. 7(2):97–110. 15158766 10.1016/j.drup.2004.01.004

[CIT0016] Qi HP, Gao XC, Zhang LQ, Wei SQ, Bi S, Yang ZC, Cui H. 2013. *In vitro* evaluation of enhancing effect of borneol on transcorneal permeation of compounds with different hydrophilicities and molecular sizes. Eur J Pharmacol. 705(1–3):20–25. 23458068 10.1016/j.ejphar.2013.02.031

[CIT0017] Rocha CR, Garcia CC, Vieira DB, Quinet A, de Andrade-Lima LC, Munford V, Belizario JE, Menck CF. 2015. Glutathione depletion sensitizes cisplatin- and temozolomide-resistant glioma cells *in vitro* and *in vivo*. Cell Death Dis. 6(4):e1727. 25880094 10.1038/cddis.2015.101 PMC4650535

[CIT0018] Schaff LR, Lassman AB. 2015. Indications for treatment: is observation or chemotherapy alone a reasonable approach in the management of low-grade gliomas? Semin Radiat Oncol. 25(3):203–209. 26050591 10.1016/j.semradonc.2015.02.008 PMC4562771

[CIT0019] Sprauten M, Darrah TH, Peterson DR, Campbell ME, Hannigan RE, Cvancarova M, Beard C, Haugnes HS, Fossa SD, Oldenburg J, et al. 2012. Impact of long-term serum platinum concentrations on neuro- and ototoxicity in cisplatin-treated survivors of testicular cancer. JCO. 30(3):300–307. 10.1200/JCO.2011.37.4025 PMC3269954 22184390

[CIT0020] Su J, Lai H, Chen J, Li L, Wong YS, Chen T, Li X. 2013. Natural borneol, a monoterpenoid compound, potentiates selenocystine-induced apoptosis in human hepatocellular carcinoma cells by enhancement of cellular uptake and activation of ROS-mediated DNA damage. PLoS One. 8(5):e63502 23700426 10.1371/journal.pone.0063502 PMC3658975

[CIT0021] Wandee J, Prawan A, Senggunprai L, Kongpetch S, Kukongviriyapan V. 2019. Metformin sensitizes cholangiocarcinoma cell to cisplatin-induced cytotoxicity through oxidative stress mediated mitochondrial pathway. Life Sci. 217:155–163. 30528773 10.1016/j.lfs.2018.12.007

[CIT0022] Wijdeven RH, Pang B, Assaraf YG, Neefjes J. 2016. Old drugs, novel ways out: drug resistance toward cytotoxic chemotherapeutics. Drug Resist Updat. 28:65–81. 27620955 10.1016/j.drup.2016.07.001

[CIT0023] Wu J, Xie J, Zhang QL, Lin ZX, Xie H, Sun B. 2016. [Research progress on meridian-guiding theory of traditional Chinese medicine]. Zhongguo Zhong Yao Za Zhi. 41(13):2428–2434. 28905564 10.4268/cjcmm20161310

[CIT0024] Xing YM, Yan XN, Guo JQ, Zhang R. 2016. Effect of borneol on the permeability of blood tumor barrier model and its mechanism study. Zhongguo Zhong Xi Yi Jie He Za Zhi. 36(6):696–702. 27491229

[CIT0025] Zhang Y, Zheng S, Zheng JS, Wong KH, Huang Z, Ngai SM, Zheng W, Wong YS, Chen T. 2014. Synergistic induction of apoptosis by methylseleninic acid and cisplatin, the role of ROS-ERK/AKT-p53 pathway. Mol Pharmaceutics. 11(4):1282–1293. 10.1021/mp400749f 24555485

[CIT0026] Zhou YD, Hou JG, Yang G, Jiang S, Chen C, Wang Z, Liu YY, Ren S, Li W. 2019. Icariin ameliorates cisplatin-induced cytotoxicity in human embryonic kidney 293 cells by suppressing ROS-mediated PI3K/Akt pathway. Biomed Pharmacother. 109:2309–2317. 30551489 10.1016/j.biopha.2018.11.108

