# Supplementary material for: Natural borneol sensitizes human glioma cells to cisplatin-induced apoptosis by triggering ROS-mediated oxidative damage and regulation of MAPKs and PI3K/AKT pathway
Source: Pharm Biol. 2019 Dec 26;58(1):72–9. doi: 10.1080/13880209.2019.1703756 (PMC6970185; doi:10.1080/13880209.2019.1703756)
Supplement: Supplemental Material [file IPHB_A_1703756_SM7719.doc]

Supporting Information for

**Natural borneol sensitizes human glioma cells to cisplatin-induced apoptosis by triggering ROS-mediated oxidative damage and regulation of MAPKs and PI3K/AKT pathway**

Wen-qiang Cao^a,b^, Xiao-qian Zhai^c^, Ji-wei Ma^d^, Xue-qi Fu^a^, Bai-song Zhao^b^, Pu Zhang^e^, Xiao-yan Fu^f^

^a^School of Life Sciences, Jilin University, Changchun, Jilin, 130012, China

^b^Zhuhai Hopegenes Medical & Phamaceutical Institute, Hengqin New Area, Zhuhai,519000, China

^c^Department of Pathology, The Second Affiliated Hospital of Shandong First Medical University, Taian, Shandong, 271000, China

^d^Department of Pathology, Shandong Provincial Hospital Affiliated to Shandong University, Jinan,250021, China

^e^Department of Cardiology, The Central Hospital of Taian, Taian, Shandong, 271000, China

^f^School of Basic Medicine, Shandong First Medical University & Shandong Academy of Medical Sciences, Taian, Shandong, 271000, China

Wen-qiang Cao and Xiao-qian Zhai contributed equally to this work.

**Running Head:** Borneol enhances cisplatin-induced apoptosis

**Corresponding authors：**

**Xiao-yan Fu**, Key Lab of Cerebral Microcirculation in Universities of Shandong, Taishan Medical University, Taian, Shandong, 271000, China, Tel: +86-538-6230027; Email: [txyfu66@163.com](mailto:txyfu66@163.com)

**Pu Zhang**, Department of Cardiology, The Central Hospital of Taian, Taian, Shandong, 271000, China, Tel: +86-18264811566; Email: [zp8198423@163.com](mailto:zp8198423@163.com)

**Figure S1.** **Cytotoxicity of cisplatin and/or NB towards HUVEC cells.** Cells were treated with 10-40 μg/mL cisplatin and/or 40 μg/mL NB for 48 h. Cell viability was determined by MTT assay after treatment. Each value represents the mean ± SD of three independent experiments. Bars with "*" indicate the *p*< 0.05.

**Figure S2. Hoechst33342/PI co-staining assay.** Cells were treated with 40 μg/mL cisplatin and 40 μg/mL NB for 48 h. Then cells were stained by Hoechst33342 (blue) and PI (red). The PI-positive cells (red) were used to label the cells necrosis. The stained cells were detected by fluorescence microscope (PI: 533/617 nm; Hoechst33342: 350/461 nm).

**Figure S3. Quantification of active-caspase-7, -9 and -3 expression.** Changes in the levels of protein expression were expressed as the percentage (%) of control. Each value represents the mean ± SD of three independent experiments. Bars with "*" or "**" indicate the *p*< 0.05 or *p*<0.01, respectively.

**Figure S4. Quantification of Ser 1981-ATM, Ser 428-ATR, Ser 15-p53, Total-p53 and Ser 139-Histone expression.** Changes in the levels of protein expression were expressed as the percentage (%) of control. Each value represents the mean ± SD of three independent experiments. Bars with "*" or "**" indicate the *p*< 0.05 or *p*<0.01, respectively.

**Figure S5. Quantification of** **Thr183-JNK, Total-JNK, Thr180-p38, Total-p38 Thr202-ERK, Total-ERK, Ser473-AKT and Total-AKT expression.** Changes in the levels of protein expression were expressed as the percentage (%) of control. Each value represents the mean ± SD of three independent experiments. Bars with "*" or "**" indicate the *p*< 0.05 or *p*<0.01, respectively.

**Figure S6. Quantification of Thr202-ERK and Ser473-AKT expression.** Changes in the levels of protein expression were expressed as the percentage (%) of control. Each value represents the mean ± SD of three independent experiments. Bars with different characters (a, b and c) are statistically different at *p*< 0.05 level.

**Figure S7. Quantification of Thr183-JNK, Thr180-p38, Thr202-ERK and Ser473-AKT expression.** Changes in the levels of protein expression were expressed as the percentage (%) of control. Each value represents the mean ± SD of three independent experiments. Bars with different characters (a, b and c) are statistically different at *p*< 0.05 level.
